# Assessing the Risk of Windborne Dispersal of *Culicoides* Midges in Emerging Epizootic Hemorrhagic Disease Virus Outbreaks in France

**DOI:** 10.1155/2024/5571195

**Published:** 2024-06-28

**Authors:** Amandine Bibard, Davide Martinetti, Albert Picado, Karine Chalvet-Monfray, Thibaud Porphyre

**Affiliations:** ^1^ Global Innovation Boehringer Ingelheim Animal Health France, Saint Priest, France; ^2^ Biostatistiques et Processus Spatiaux UMR 0546 INRAE, Avignon, France; ^3^ Epidémiologie des Maladies Animales et Zoonotiques UMR EPIA INRAE Université Clermont Auvergne, VetAgro Sup, Saint-Genès-Champanelle, France; ^4^ Laboratoire de Biométrie et Biologie Évolutive UMR 5558 CNRS Université Claude Bernard Lyon 1, VetAgro Sup, Villeurbanne, France

## Abstract

The epizootic hemorrhagic disease virus (EHDV) is a novel emerging threat for the European livestock sector. First detected in Sardinia and southern Spain at the end of 2022, this transboundary disease emerged in France in September 2023 despite restrictions on animal movement and enhanced surveillance protocols. Although virus spread is believed to be mediated by the dispersal of *Culicoides* vectors by the wind, prediction is difficult due to the large number of meteorological parameters that must be considered. Using simulations of atmospheric trajectories, we developed a model to investigate the long-distance dispersal risk zone of *Culicoides* in Europe, starting from different source zones. Our model predicted with good sensitivity the newly EHDV-infected areas in France over a period of 5 weeks after its first introduction in the country. Prospectively, we predicted that the midge dispersal zone of early 2024 could expand toward most of the western half of France and could sporadically reach new countries under favorable spring conditions. The wind dispersal risk maps provided are intended to support better preparedness and response to *Culicoides*-borne diseases.

## 1. Introduction

Epizootic hemorrhagic disease (EHD) is a hemorrhagic fever affecting both domestic and wildlife ruminants and listed as notifiable disease since 2008 [1]. The disease is induced by the epizootic hemorrhagic disease virus (EHDV), member of the *Orbivirus* genus, like Bluetongue virus (BTV) and African horse sickness virus (AHSV). Seven different serotypes of the virus have been officially recognized so far (EHDV-1-2, 4-8) [2, 3].

Originally, the disease was identified in the south of USA in 1955 [4] and expanded toward northern states to be considered endemic nowadays for serotypes 1, 2, and 6 [5, 6]. While the disease was mostly pathogenic in wild ruminants in the USA, outbreaks of other EHDV variants/serotypes reported in Asia (EHDV-2 variant in Japan), in the middle east (EHDV-7 in Israel, EHDV-6 in Turkey and Oman [7]) and in north of Africa (EHDV-6 in Tunisia, Morocco, Algeria [7]) were associated with clinical signs in domestic cattle [5]. Between 2003 and 2017, the overseas territories of France (Reunion Island [8], Martinique, Mayotte, and Guiana [9]) also experienced cattle outbreaks of EHDV-1, -2, and -6.

EHDV-8 was originally identified in Australia in 1982 [5] and had never been reported in any other country until the end of 2021 when Tunisia identified clinical cases in cattle [10]. The year after, in October 2022, Sardinia, Sicily, and Spain also reported cases of this serotype [11, 12], constituting the official incursion of the disease into the European continent. The disease subsequently spread northwards across the Iberian Peninsula and reached the southern border of France in September 2023 [13]. The lack of an EU-licensed vaccine against this disease/serotype [1] leaves Europe vulnerable to subsequent spread, which represents an emerging threat to both livestock and wildlife in free countries. Predictions of where and when further outbreaks could occur are critical to enhance preparedness and response against such incursions.

EHDV is transmitted by hematophagous midges, *Culicoides* spp., present worldwide including in Europe [14]. The two most widespread *Culicoides* species in France [15], *C. obsoletus* and *C. scoticus*, have been shown to be susceptible to EHDV, at least to the serotypes 6 (EHDV-6) [16] and 8 (EHDV-8) [17]. Due to their very small size (1–2.5 mm) [18], *Culicoides* spp. can be easily uplifted in the air and subsequently dispersed in the air by the prevailing winds. Using the capture–recapture technique, they have been found within a few kilometers from their breeding sites on animal farms [19] but strong evidence exists that adult midges can be passively dispersed by wind over long distances under favorable conditions [20, 21, 22, 23]. They were also identified at high altitude [24, 25]. Dispersal by the wind has been identified as the most likely route of introduction for *Culicoides*-borne diseases in many countries, for example, EHDV-2 in British Columbia [26], BTV in Australia [27], and Schmallenberg virus (SBV) in Ireland [28]. With regard to EHDV-8, its introduction into Sardinia and Sicily strongly suggests a similar windborne pathway from Tunisia as was evidenced for BTV-3 in 2017 [29].

Although considered important, the windborne dispersal of insects is extremely challenging to monitor since it is technically unfeasible to sample large volumes of air at the needed frequency, while predicting potential dispersal can only rely on modeling approaches based on atmospheric simulations.

In recent years, the use of atmospheric dispersion models to mimic the atmospheric trajectories of flying vectors has been on the rise. Among these, HYSPLIT [30], an open-source model originally developed to simulate the trajectories of inert particles in the atmosphere, has been increasingly used to assess dispersal of fungus spores [31], *Culicoides* spp. [27, 29, 32], and other flying insect [33]. This atmospheric model has been mostly used retrospectively to assess the most likely source of a virus incursion [29], but never prospectively in the context of early disease management. Moreover, the complex management of the HYSPLIT model and wind data [34] remains a limitation to its rapid and efficient use in an urgent context of disease emergence at the scale of Europe. However, new tools have recently been proposed to simplify the simulation of air-mass trajectories and their use in the context of epidemic surveillance [35].

Here, we aim to estimate the magnitude of *Culicoides* dispersal by the wind in the context of the emergence of EHDV-8 in France in 2023. To do so, we developed a wind connectivity matrix, based on simulations from an atmospheric model (HYSPLIT) that has been adapted to *Culicoides* survival conditions, to evaluate when and how often two locations anywhere in Europe could be connected by wind only. To validate our approach, we also investigate retrospectively the spatial matching between risk predictions and subsequent disease outbreaks. Finally, we aim to explore prospectively with the same methodology, the potential geographic extent of the disease in 2024 at the restart of the *Culicoides* season.

## 2. Materials and Methods

### 2.1. HYSPLIT Atmospheric Trajectories and Wind-Connectivity Matrix

We defined the study grid as a spatial area that includes European countries, north Africa, and the Balkans, divided into 32,292 grid cells of resolution 0.25° (~25 × 25 km) after removal of points below ground level (sea/lakes) (Figure S1).

Starting from the centroid of each grid cell, we simulated atmospheric trajectories using the HYSPLIT model [30] (HYbrid Single-Particle Lagrangian Integrated Trajectory, version 5) in a forward mode. Each atmospheric trajectory was initiated twice a day, at sunrise and sunset (corresponding to a peak of midges' activity and favorable time for take-off in ascendant airstreams [36, 37]), over a period of 36 weeks (from mid-March to mid-November, i.e., from week 11 to week 46), and for each year between 2020 and 2023. A minimum altitude of 50 m above ground level was established at the beginning of each trajectory. Along each simulated trajectory, meteorological and atmospheric variables were recorded hourly for a period of 24 hr. Every point along the trajectory where the temperature was above 10°C and the altitude of the trajectory was below the height of the planetary boundary layer (PBL) was considered as a potential deposition point. The temperature threshold reflects the minimum temperature of midge activity [38, 39] and trajectories experiencing below it were automatically stopped, under the assumption that midges within that air mass could not survive or remain active. The altitude condition ensures that the trajectory is within the lowest, most turbulent layer of the atmosphere, the one in contact with the ground, and hence midges may be deposited [25, 40], instead of following the laminar flow above the PBL. The atmospheric trajectory, if not stopped by temperature or altitude limits, was stopped after a maximum dispersal time of 24 hr, corresponding to a median of maximum flight time for *Culicoides* used in previous studies (12–48 hr) [22, 28, 41, 42, 43].

As a result of HYSPLIT simulations, a wind-connectivity matrix was built to list the number of times a destination grid cell *j* was reached at least once from a source grid cell *i* for each day of the study period, with *i* and *j* being any grid cell the study grid.

### 2.2. Zone of EHDV Infection in France

Details of 220 reported events of EHDV outbreak occurring in France between 1 September and 1 December 2023, including geographic positions and start date, were retrieved from the WAHIS database of the World Organization for Animal Health [44]. To ensure that the wind could transport potentially EHDV-infected *Culicoides*, grid cells were identified where at least one outbreak of EHDV occurred and considered as source grid cells *i* in the wind-connectivity matrix.

**Figure 1:**
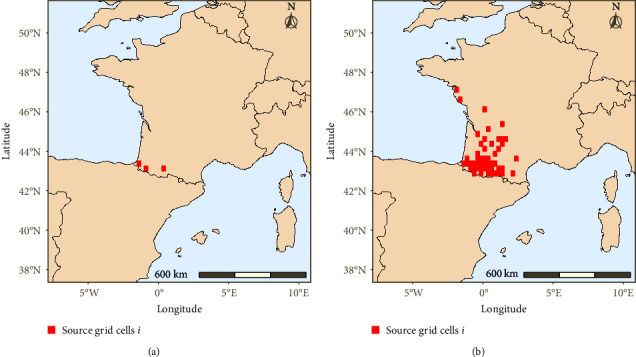
Definitions of the two source zones *Z*_1_ and *Z*_2_ composed of source grid cells *i* from where atmospheric trajectories were initiated: (a) “index source zone *Z*_1_” includes three grid cells where the first three EHDV outbreaks occurred on September 4, 8, and 9 2023; (b) “secondary source zone *Z*_2_” includes 50 grid cells where EHDV outbreaks occurred between September 4 and November 15.

For the purpose of this study, we defined two different infected source zones *Z*_*x*_ that encompass several grid cells *i* according to disease situations in France (Figure 1): (1) the “index source zone” *Z*_1_, which includes the three grid cells *i* with the first reported EHDV-8 outbreaks in France that were believed to be infected on the 4, 8, and 9 September (week 36), (2) the “secondary source zone” *Z*_2_, which includes the 50 grid cells of the 220 EHDV-8 outbreaks reported to WAHIS before 1 December 2023 and believed to be infected between 4 September and 15 November 2023.

### 2.3. Probability of Windborne Dispersal and Risk Maps

From the wind-connectivity matrix, we extracted, for each source zone *Z*_*x*_ considered, all the destination grid cells *j* that were reached at least once from any of the source grid cell *i* within 24 hr. By summing the number of grid cells *i* comprised in each source zone *Z*_*x*_ and over a specific period *T*_*y*_ in days, a frequency of arrival was calculated for each destination grid cell *j* and was used as a proxy of a daily probability of long-distance dispersal (*H*_*j*(*Z*_*x*_, *T*_*y*_)_), as follows:

(1) $$\begin{array}{c} H_{j\left(Z_{x},T_{y}\right)}=\frac{1}{s_{x}t_{m}\alpha }\sum_{i=1}^{s_{x}}\sum_{t=1}^{t_{m}}D_{ijt}, \end{array}$$

where *H*_*j*(*Z*_*x*_, *T*_*y*_)_ is the average daily probability of a given destination cell *j* over a period *T*_*y*_ of *t*_*m*_ days, for a midge to be airborne dispersed from source zone *Z*_*x*_; *D*_*ijt*_ is the number of connections between the source cell *i* and the destination cell *j* at day *t*; *s*_*x*_ is the number of source grid cells *i* in the source zone *Z*_*x*_; *t*_*m*_ the total number of days in the period *T*_*y*_, with *T*_1_for a period of 1 week (*t*_*m*_ = 7 days) and *T*_5_ for a period of 5 weeks (*t*_*m*_ = 45 days); and *α* is the maximal number of possible destinations reached in a day from a given source cell *i*, such as *α* = 48 (24 potential deposition spots per trajectory, two trajectories started per day).

The variation of *H*_*j*(*Z*_*x*_, *T*_*y*_)_ in time and in space was interpreted as a risk of *Culicoides* dispersal in each destination cell *j*, knowing the source zone and the period, and was mapped accordingly.

### 2.4. Retrospective Scenario 1 and Model Validation

In Scenario 1, we applied the model in a retrospective situation, considering the index source zone *Z*_1_ (the first EHDV outbreaks in France started in early September 2023—week 36) and investigating the risk of dispersal for the five subsequent weeks (week 37 to week 41, from mid-September to mid-October), based on meteorological data of 2023 only. The analysis was chosen for a period of 5 weeks given the variable lifespan of *Culicoides* spp. between 2 and 6 weeks depending on the environment [45], and a virus incubation period of 3 days in cattle after vector bite [46]. The extrinsic incubation period (EIP) of EHDV in *Culicoides* spp. (5 days or shorter [47, 48]) was not considered because it was assumed that the vector population in the source zone was already infectious and because the impact of the EIP (addition of 1 week to the time period) on the spatial distribution of risk was found to be negligible (Figures S3 and S4).

We validated the model by estimating the accuracy of our risk predictions averaged between week 37 and week 41 (*H*_*j*(*Z*_1_,*T*_5_)_) in France, over the locations of the 186 EHDV outbreaks reported in the country within the same period (between 11 September and 15 October 2023). Outbreaks that were reported outside of our study grid were excluded from the analysis, as well as those emerging into a source grid cell *i* as we assumed that they were generated from local dispersion. For the purpose of the analysis, the risk prediction *H*_*j*(*Z*_1_,*T*_5_)_ was retrieved for each outbreak and an empirical cumulative distribution of *H*_*j*(*Z*_1_,*T*_5_)_ was plotted. The presence and absence of at least one reported EHDV outbreak in each cell *j* were counted and their respective proportion was plotted in each risk interval of *H*_*j*(*Z*_1_,*T*_5_)_.

The predictive performance of our model on the presence/absence of an EHDV outbreak was measured using the area under the curve (AUC) of a receiver operating characteristic (ROC) curve using the *pROC* [49] package in the statistical environment R [50]. Here, we considered the predictive performance of our model as good if AUC > 0.7, very good if AUC > 0.8, and excellent if AUC > 0.9.

We further characterized the performance of the model by computing the true positive ratio (TPR) and the false positive ratio (FPR) for different risk threshold values *θ*. Here, we defined the TPR as the proportion of grid cells *j* with *H*_*j*(*Z*_1_,*T*_5_)_ ≥ *θ* that correctly predicted the presence of at least one outbreak, while the FPR was defined as the proportion of grid cells with *H*_*j*(*Z*_1_,*T*_5_)_ ≥ *θ* with no record of EHDV outbreak. It should be noted that TPR is equivalent to the sensitivity of a diagnostic test, whereas FPR is equivalent to 1 − specificity, and both can be extrapolated upon the shape of the previously computed ROC curve [51]. In this analysis, we defined three situations in the upper left part of the ROC curve where the risk threshold *θ* either maximizes only the TPR (A), minimizes only the FPR (C), or optimizes the values of both TPR and FPR (B). For each situation, we also calculated the size of the corresponding risk zones in France (i.e., the surface area covered by grid cells with *H*_*j*(*Z*_1_,*T*_5_)_ ≥ *θ*) and its proportion in regard of the entire risk zone (i.e., the surface area covered by grid cells with *H*_*j*(*Z*_1_,*T*_5_)_ > 0).

### 2.5. Prospective Scenario 2

In Scenario 2, we investigated the risk of windborne dispersal of infected *Culicoides* in a prospective manner. We defined the secondary source zone *Z*_2_ as the EHDV-infected area reported early December 2023 (last available data) considering three main assumptions in this area: (1) it will remain roughly stable during the 2023 winter diapause of midges, (2) the virus is likely to overwinter due to a limited but sufficient number of viraemic cattle, which could be the source of infection for new adult midges feeding inside the animal housing [52], (3) the outside midge activity, principally for Obsoletus complex, could restart in 2024 as early as mid-March in France [53]. The model was then used to predict the windborne dispersal zone at risk for the upcoming year, from mid-March to mid-April (5 weeks from week 11 to week 15), considering the secondary source zone *Z*_2_. This scenario uses the HYSPLIT data averaged over the 4-year period from 2020 to 2023 to account for the variability of meteorological conditions over the years.

## 3. Results

### 3.1. Wind-Dispersal Risk Zone from Index EHDV Outbreaks (Scenario 1)

**Figure 2:**
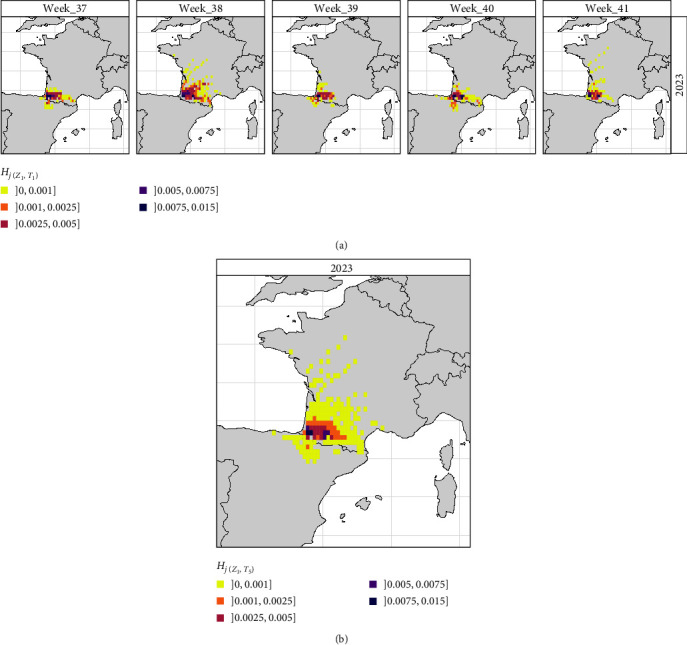
Spatial distribution of (a) *H*_*j*(*Z*_1_,*T*_1_)_, the daily probability averaged by week (*T*_1_) between week 37 and week 41 (mid-September to mid-October), and (b) *H*_*j*(*Z*_1_,*T*_5_)_, the daily probability averaged over the 5 weeks (*T*_5_) of the same period; *H*_*j*(*Z*_1_,*T*_1_)_ and *H*_*j*(*Z*_1_,*T*_5_)_ are both computed here considering the index source zone *Z*_1_ (the first three EHDV outbreaks started in France in week 36) and the meteorological conditions of 2023 (scenario 1).

Areas with an average daily probability of wind dispersal *H*_*j*(*Z*_1_,*T*_1_)_ > 2.5 × 10^−3^ remain mostly restricted to the southwest of France close to the source zone (Figure 2(a)), except in week 38 during which the risk area of wind dispersal extended both northward and eastward. Areas with lower predicted daily probabilities were widely observed throughout the southwest region of France (Figure 2(a)), along the Pyrenees Mountains from their Atlantic to Mediterranean endpoints, and toward the northern direction, up the Gironde Estuary or, exceptionally, up to the Loire Valley. Potential wind dispersal events into north of Spain were also observed in weeks 37, 39, and 40, particularly in the Spanish Basque Country and at the north of Navarra, Huesca, and Zaragoza regions.

During the 5 weeks after the emergence of EHDV, the predicted zone with the highest risk of wind dispersal, that is, where *H*_*j*(*Z*_1_,*T*_5_)_ > 10^−3^, covers an area of ~ 25,000 km^2^ (i.e., 40 grid cells *j*), mostly located north-east of the source zone (Figure 2(b)). Although showing *H*_*j*(*Z*_1_,*T*_5_)_ < 10^−3^, incursions along the western border, toward the center of France, and toward the north of Spain were also possible.

### 3.2. Model Validation (Scenario 1)

**Figure 3:**
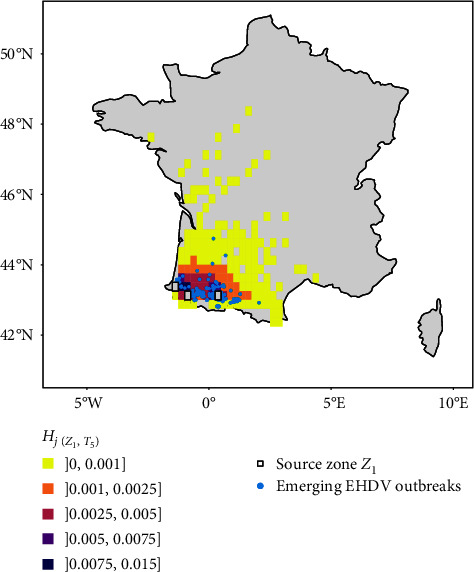
Spatial overlay between risk predictions (*H*_*j*(*Z*_1_,*T*_5_)_, the daily probability averaged over 5 weeks from week 37 to week 41) and the 186 EHDV outbreaks emerging within the same period of time in France. *H*_*j*(*Z*_1_,*T*_5_)_ is computed here considering the index source zone *Z*_1_ (the first three EHDV outbreaks started in France in week 36), and the meteorological conditions of 2023 (scenario 1).

**Figure 4:**
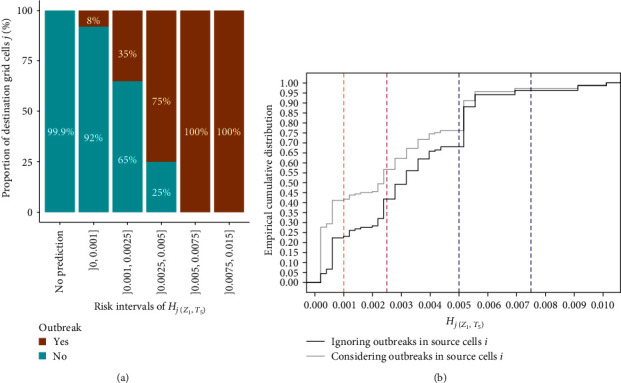
Validation plots: (a) proportion of destination grid cells *j* where at least one emerging EHDV outbreak occurred (“yes”) or did not occur (“no”) in each risk interval (*H*_*j*(*Z*_1_,*T*_5_)_). Destination grid cells equivalent to index source grid cells were not considered; (b) empirical cumulative distribution of emerging outbreaks according to their associated risk predictions (*H*_*j*(*Z*_1_,*T*_5_)_). The dashed color lines represent the upper limits of the risk intervals.

Among the 186 EHDV outbreaks reported between W37 and W41, two (1.1%) occurred outside the study grid on the Atlantic coast and 49 (26.3%) occurred in cells of the index source grid (Figure 3). All remaining 135 outbreaks, except one (99.9%) were found located in a predicted destination cell with *H*_*j*(*Z*_1_,*T*_5_)_ > 0 (Figures 3 and 4). The only outbreak that occurred in a cell without prediction (*H*_*j*(*Z*_1_,*T*_5_)_ = 0) was located close to the Pyrenees mountains.

Figure 4(a) shows that a high proportion of destinations with a very low-risk interval (]0,0.001]) were places where no outbreaks occurred, while all destinations with the highest risk (]0.0075,0.015], ]0.005,0.0075]) had at least one EHDV outbreak. The proportion of destinations with at least one EHDV outbreak was still high (75%) within the risk interval ]0.0025,0.005], and then decreased to 35% and 8% in the lowest risk intervals ]0.001, 0.0025] and ]0, 0.001], respectively (Figure 4(a)). The exact number of grid cells *j* in each risk interval is detailed in Figure S2. The risk zone with a predicted risk *H*_*j*(*Z*_1_,*T*_5_)_ > 10^−3^ encompassed 76.9% of the reported EHDV outbreaks (*n* = 103), while 31 outbreaks (23.1%) were found in cells showing *H*_*j*(*Z*_1_,*T*_5_)_ < 10^−3^ (Figure 4(b)). However, the proportion of outbreaks in risk zone *H*_*j*(*Z*_1_,*T*_5_)_ < 10^−3^ would be higher (41.7%) if considering the total number of outbreaks, including those that occurred within the source grid cells *i* (Figure 4(b)).

**Figure 5:**
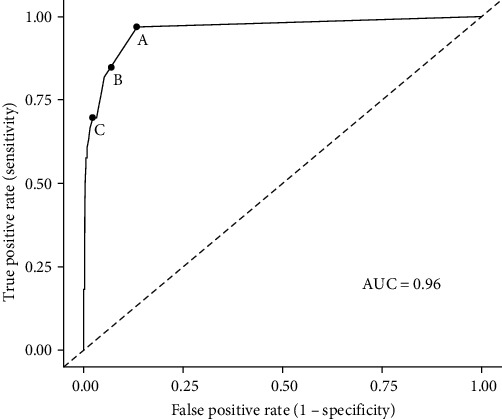
Receiver operating characteristic (ROC) curve characterizing the global performance of the model. Model performance was measured by the AUC (area under the curve). A, B, and C refer to three situations on the upper left part of the ROC curve where the risk threshold *θ* either maximizes only the true positive rate (A), minimizes only the false positive rate (C), or optimizes values of both TPR and FPR (B).

The performance of our predictions appears satisfactory, with an AUC of 0.96, highlighting an excellent predictive capacity of our model (Figure 5). The TPR of the risk predictions was maximized for a threshold value of *θ* = 10^−4^ (situation A), showing a sensibility of 97% but a FPR of 13.3% (Table 1). On the contrary, the FPR of the risk predictions was minimized for a *θ* = 9 × 10^−4^ (situation C), limiting the FPR to 2.2% but decreasing the TPR to 69.7%. However, both TPR and FPR were optimized (situation B) when the risk threshold *θ* = 3 × 10^−4^. If surveillance activities were to be implemented in grid cells where *H*_*j*(*Z*_1_,*T*_5_)_ ≥ *θ*, surveillance activities would be implemented in areas greater than 103,000, 61,000, and 28,000 km^2^ for situations A, B, and C, respectively. These represent 100%, 58.8%, and 27.9% of the full risk zone in France (103,125 km^2^), which would correspond to nearly 18% of the total surface area of continental France and Corsica.

### 3.3. Prospective Wind Dispersal Risk Zone for 2024 (Scenario 2)

**Figure 6:**
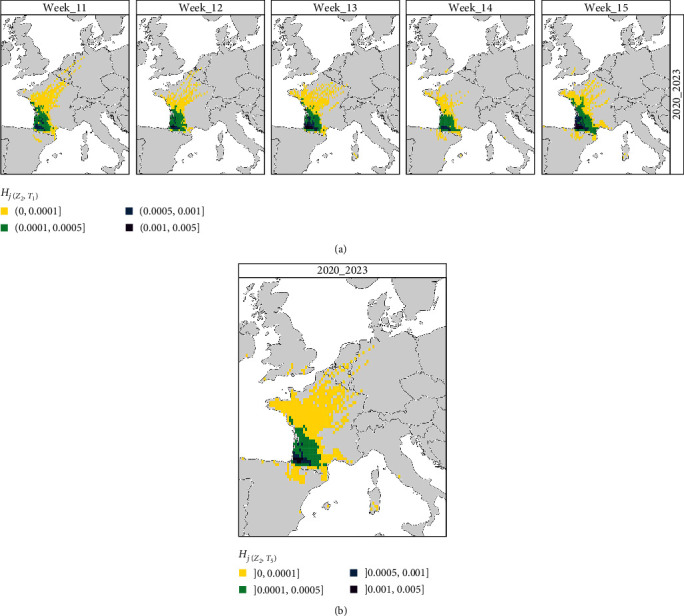
Spatial distribution of (a) *H*_*j*(*Z*_2_,*T*_1_)_, the daily probability averaged by week (*T*_1_—from W11 to W15; from mid-March to mid-April) of long-distance dispersal, and (b) *H*_*j*(*Z*_2_,*T*_5_)_, the daily probability averaged over 5 weeks (*T*_5_−from W11 to W15; from mid-March to mid-April) of long-distance dispersal. Both *H*_*j*(*Z*_2_,*T*_1_)_ and *H*_*j*(*Z*_2_,*T*_5_)_ are both computed here considering the secondary source *Z*_2_ (the whole EHDV-infected area in early December 2023 in France) and the 4-year period 2020–2023.

When projecting the risk of wind dispersal during the 5-week period from mid-March, areas that showed a predicted probability *H*_*j*(*Z*_2_,*T*_5_)_ > 10^−4^ covered most of the south-western quarter of France, from the Atlantic extremity toward the eastern Mediterranean and north along the Atlantic border (Figure 6(b)). This risk zone is well preserved over the 5-week period, although in week 15, the risk zone with *H*_*j*(*Z*_2_,*T*_5_)_between 10^−4^ and 5 × 10^−4^ (green color) is more extended to the north and the south-east compared to the previous weeks (Figure 6(a)).

On average, the full risk zone, including probability *H*_*j*(*Z*_2_,*T*_5_)_ < 10^−4^, covers three quarters of the country, excluding the Massif Central and the south-eastern quarter of the country (Figure 6(b)). Destinations outside France were also reached sporadically in the UK and Ireland toward the north, and in Belgium, the Netherlands, and Germany toward the north-east. It is interesting to note that southerly incursions into northern Spain remain rare and spatially limited despite the proximity of the source zone. Extreme south incursion into the Balearic Islands, southern Sardinia, and the western border of Italy has also been observed once or twice during the period, but never before week 13 (end March).

## 4. Discussion

In retrospective scenario 1, our modeling results showed that the high-risk zone (*H*_*j*_ > 10^−3^) of wind-dispersal from the first outbreaks in France remains mostly limited in the southwest corner of the country but with weekly northern incursions up to the estuary of the “Gironde” region or eastern incursions along the Pyrenees mountains (Figures 2 and 3). The model also identified sporadic dispersal toward the north of Spain, illustrating a wind connection between the two countries, as evidenced in the past for BTV-1, also transmitted by *Culicoides* vectors [54]. However, wind connection was rare and only possible at the western endpoint of the Pyrenees, with most of the mountains acting as an orographic barrier.

The predictions of scenario 1 fitted well with the locations where the EHDV outbreaks occurred: the full risk zone in France (cells with *H*_*j*(*Z*_1_,*T*_5_)_ > 0 excluding the source ones) captured 99.9% of the EHDV outbreaks (Figure 3) and the high-risk predicted cells (with *H*_*j*(*Z*_1_,*T*_5_)_ > 5 × 10^−3^) all reported EHDV outbreaks (Figure 4). However, 23.1% of the EHDV outbreaks also occurred in areas with low-risk *H*_*j*(*Z*_1_,*T*_5_)_ < 10^−3^ (Figure 4(b)). The only false negative case observed was located close to the Pyrenees, suggesting an underestimation of the risk in mountainous areas, probably due to rugged terrain that limits wind penetration in these areas.

ROC analysis confirmed the excellent ability of our model to predict reports of EHDV-8 outbreaks in France, with an AUC value of 0.96 (Figure 5) and a very good sensitivity of 97% for *H*_*j*(*Z*_1_,*T*_5_)_ ≥ 10^−4^. However, at this risk threshold, the risk zone covers a very large area of ~18% of France and includes at-risk locations incorrectly identified by the model (false positive rate of 13.3%, Table 1). Depending on the surveillance strategy and the relative costs associated with false positives, it could be considered to limit the surveillance zone by adopting lower risk thresholds. Reducing the risk zone by ~40% and ~72% (risk thresholds of 3 × 10^−4^ and 9 × 10^−4^, respectively) could significantly reduce the false positive rate (−6.4% and −11.1% loss of FPR, respectively), but, on the other hand, would affect the ability of the model to predict the presence of outbreaks (−12.2% and −27.3% loss of TPR, respectively).

It is worth noting that a significant proportion of reported EHDV-8 outbreaks were excluded from our validation analysis. First, only a few (1.1%) fell outside the study grid due to the 0.25° resolution, which was not precise enough to include certain areas near the coasts. Second, 26.3% (49 out of 186) of the outbreaks fell into the three source grid cells, where our predicted risk of wind dispersal was either null or very low. This finding suggests a local spread due to *Culicoides* that did not enter the wind stream and generated new outbreaks within a short-range area. In our model, atmospheric simulations begin at a high enough altitude (50 m) assuming that midges are already within the air mass. It does not consider those that remain close to the ground, therefore, underestimating this local dispersion. Taking into account the outbreaks that occurred in the source grid cells impaired the performance of the model (Figure 4(b)). This result illustrates that the model should be used primarily to estimate long-range dispersal but remains inadequate for short-range dispersal.

Routes other than the wind dispersal of *Culicoides* could have played a role in the emergence of EHDV-8 outbreaks and could have biased our analysis. The movement of infected animals and the spillover from wildlife are recognized pathways for the spread of EHDV [2]. However, their impact, if confirmed, might be limited given the restrictions on animal movements within the 150 km regulated area [55] and the low number of cases reported so far in European wildlife populations [12, 56].

Considering the prospective scenario 2, higher risk destinations (*H*_*j*_ > 10^−4^) were mostly localized within the western quarter of France, and the risk area did not extend much beyond the source zone *Z*_2_, except from mid-April (Week 15) when the temperature is getting warmer. Regarding the lowest probabilities (*H*_*j*_ < 10^−4^), the risk area is much broader and includes more than the western half of France. The south-eastern quarter of France was not exposed to a risk, probably due to the Massif central acting as an orographic barrier, similarly to the role played by the Pyrenees in the south. The dispersal zone could also sporadically reach free countries despite their long distance from the source, both over vast bodies of water (such as UK and Ireland toward north, and the Balearics and continental Italy toward south) and over land (such as Belgium, Germany, and the Netherlands). However, those events could be considered rare and highly dependent on meteorological conditions. It is worth noting that the assumptions underlying this scenario 2 (principally virus overwintering and *Culicoides* activity onset from mid-March) are uncertain and highly depend on the unknown upcoming weather. Analyzing the wind connectivity matrix over four different years in this scenario allowed us to capture the main global pattern, although this approach could minimize or hide sporadic dry and windy events, more frequently observed in the context of climate change [57]. As our model cannot predict upcoming weather, it appears important to keep enriching the wind-connectivity matrix for subsequent years, to adapt as much as possible the predictions according to the meteorological conditions.

Globally, the wind dispersal zones provided in both scenarios appear to follow a preferred direction from south to north/northwest, influenced by wind direction, wind speed, topography, and temperatures. This highlights the anisotropic nature of the wind, which contrasts with the usual radius surveillance zone established around infected outbreaks. The extent of the predicted risk zone exceeded the usual 150 km distance to the north and was more limited to the south.

In our methodological approach using a wind connectivity matrix, we assumed that the disease introduction risk into a new area can be inferred from the risk of *Culicoides* dispersal by the wind. However, the “episystems”, i.e., the virus-host–vector-environment relationships that trigger the transmission or emergence of arboviruses, are complex, site-specific, and can change over time [58]. We therefore acknowledge several limitations in our approach. First, the number of *Culicoides* that are present and infectious in each grid cell *i*, uplifted in the air mass and subsequently transported, is not estimated. This number may vary greatly from grid cell to another and in time, depending on the prevalence of the disease, the abundance of the vector, and the local temperature, which triggers the disease dynamic in the insect population. However, our model assumes a minimal and homogeneous number in each source cell to allow a potential arrival at each deposition spot. Second, the model does not account for the presence of susceptible hosts at the destination. This implies that infected *Culicoides* can potentially reach a destination but will not be able to transmit the disease at the next blood meal. These limitations need to be acknowledged when communicating the risk results, and particularly if uncertainty remains about the episystems in place at source (as for scenario 2) and destinations.

Despite these limitations, our model provides useful maps of wind dispersal to raise awareness of the potential risk of vector incursion. As the wind-connectivity matrix can be used considering different sources of infection, it can be envisioned to apply it in many different situations (other sources of infection, other *Culicoides*-borne diseases). These predictions then constitute a critical parameter that can be included afterward within a complete risk assessment framework to assess with more precision the risk of disease introduction.

In conclusion, our model predicted well the locations of EHDV outbreaks due to long-range wind dispersal of *Culicoides*, but underestimated short-range dispersal, which accounted for 26% of outbreaks. Despite limitations and high uncertainties in future meteorological conditions, the model provides valuable insights into precise risk zones of dispersal adapted to local environmental conditions on a European scale. These findings can be used to raise awareness and guide future risk assessment frameworks, making them a useful tool for the management and prevention of future *Culicoides*-borne virus outbreaks.

## Figures and Tables

**Table 1:** Further characteristics of the predictive performance of the model in three situations.

Situations	*θ*	TPR (%)	FPR (%)	Area (km^2^)	Proportion (%)
A	1 × 10^−4^	97	13.3	103,125	100
B	3 × 10^−4^	84.8	6.9	60,625	58.8
C	9 × 10^−4^	69.7	2.2	28,750	27.9

The true positive rate (TPR) and the false positive rate (FPR) were computed for each situations A, B, and C corresponding to different values of risk threshold *θ*. The surface area (cells with *H*_*j*(*Z*_1_,*T*_5_)_ ≥ *θ*) and its proportion regarding the full risk surface (cells with *H*_*j*(*Z*_1_,*T*_5_)_ > 0) is also provided.

## Data Availability

The subsets of the full European wind-connectivity matrix that support our analysis are openly available in data INRAE repository within the dataverse “Experimental—Observation—Simulation,” with the following reference link: doi:10.57745/X9FWAG. Other data can be available upon request from the corresponding author.

## Supplementary Materials

Supplementary Materials Figure S1 shows the spatial limits of the full study grid (A) and the division into 0.25° grid cells for France (B); Figure S2 shows the number of grid cells in France in each risk interval (scenario 1); Figures S3 and S4 illustrate the impact of adding 1 week to the time period (6 weeks) on risk maps for scenarios 1 and 2, respectively.

## References

[1] Jiménez-Cabello L., Utrilla-Trigo S., Lorenzo G., Ortego J., Calvo-Pinilla E. (2023). Epizootic hemorrhagic disease virus: current knowledge and emerging perspectives. *Microorganisms*.

[2] EFSA Panel on Animal Health and Welfare (AHAW) (2009). Scientific opinion on epizootic hemorrhagic disease. *EFSA Journal*.

[3] Anthony S. J., Maan S., Maan N. (2009). Genetic and phylogenetic analysis of the outer-coat proteins VP2 and VP5 of epizootic haemorrhagic disease virus (EHDV): comparison of genetic and serological data to characterise the EHDV serogroup. *Virus Research*.

[4] Shope R. E., MacNamara L. G., Mangold R. (1960). A virus-induced epizootic hemorrhagic disease of the virginia white-tailed deer (*Odocoileus virginianus*). *The Journal of Experimental Medicine*.

[5] Rivera N. A., Varga C., Ruder M. G. (2021). Bluetongue and epizootic hemorrhagic disease in the United States of America at the wildlife–livestock interface. *Pathogens*.

[6] Allison A. B., Goekjian V. H., Potgieter A. C. (2010). Detection of a novel reassortant epizootic hemorrhagic disease virus (EHDV) in the USA containing RNA segments derived from both exotic (EHDV-6) and endemic (EHDV-2) serotypes. *Journal of General Virology*.

[7] Savini G., Afonso A., Mellor P. (2011). Epizootic haemorragic disease. *Research in Veterinary Science*.

[8] Cêtre-Sossah C., Roger M., Sailleau C. (2014). Epizootic haemorrhagic disease virus in Reunion Island: evidence for the circulation of a new serotype and associated risk factors. *Veterinary Microbiology*.

[9] Viarouge C., Lancelot R., Rives G. (2014). Identification of bluetongue virus and epizootic hemorrhagic disease virus serotypes in French Guiana in 2011 and 2012. *Veterinary Microbiology*.

[10] Sghaier S., Sailleau C., Marcacci M. (2023). Epizootic haemorrhagic disease virus serotype 8 in Tunisia, 2021. *Viruses*.

[11] Lorusso A., Cappai S., Loi F. (2022). First detection of epizootic haemorrhagic disease virus in the European Union, Italy-2022.

[12] Portanti O., Thabet S., Abenza E. (2023). Development and validation of an RT-qPCR for detection and quantitation of emerging epizootic hemorrhagic disease virus serotype 8 RNA from field samples. *Journal of Virological Methods*.

[13] Zientara S., Sailleau C., Dujardin P., Bréard E., Vitour D. (2024). Émergence de la maladie hémorragique épizootique en France en 2023: impacts et perspectives futures. *Virologie*.

[14] Wint G. R. W., Balenghien T., Berriatua E. (2023). VectorNet: collaborative mapping of arthropod disease vectors in Europe and surrounding areas since 2010. *Eurosurveillance*.

[15] Balenghien T., Alexander N., Arnþórsdóttir A. L. (2020). VectorNet data series 3: *Culicoides* abundance distribution models for europe and surrounding regions. *Open Health Data*.

[16] Federici V., Ippoliti C., Goffredo M. (2016). Epizootic haemorrhagic disease in Italy: vector competence of indigenous *Culicoides* species and spatial multicriteria evaluation of vulnerability. *Veterinaria Italiana*.

[17] Quaglia M., Foxi C., Satta G. (2023). *Culicoides* species responsible for the transmission of Epizootic Haemorrhagic Disease virus (EHDV) serotype 8 in Italy. *Veterinaria Italiana*.

[18] Mellor P. S., Boorman J., Baylis M. (2000). *Culicoides* biting midges: their role as arbovirus vectors. *Annual Review of Entomology*.

[19] Sanders C. J., Harrup L. E., Tugwell L. A., Brugman V. A., England M., Carpenter S. (2017). Quantification of within- and between-farm dispersal of *Culicoides* biting midges using an immunomarking technique. *Journal of Applied Ecology*.

[20] Sellers R. F., Maarouf A. R. (1989). Trajectory analysis and bluetongue virus serotype 2 in Florida 1982. *Canadian Journal of Veterinary Research*.

[21] Ducheyne E., Deken R. D., Bécu S. (2007). Quantifying the wind dispersal of *Culicoides* species in Greece and Bulgaria. *Geospatial Health*.

[22] Burgin L. E., Gloster J., Sanders C., Mellor P. S., Gubbins S., Carpenter S. (2013). Investigating incursions of Bluetongue Virus using a model of long-distance *Culicoides* biting midge dispersal. *Transboundary and Emerging Diseases*.

[23] Sedda L., Rogers D. J. (2013). The influence of the wind in the Schmallenberg virus outbreak in Europe. *Scientific Reports*.

[24] Sanders C. J., Selby R., Carpenter S., Reynolds D. R. (2011). High-altitude flight of *Culicoides* biting midges. *Veterinary Record*.

[25] Braverman Y., Chechik F. (1996). Air streams and the introduction of animal diseases borne on *Culicoides* (Diptera, Ceratopogonidae) into Israel. *Revue Scientifique et Technique de l’OIE*.

[26] Sellers R. F., Maarouf A. R. (1991). Possible introduction of epizootic hemorrhagic disease of deer virus (serotype 2) and bluetongue virus (serotype 11) into British Columbia in 1987 and 1988 by infected *Culicoides* carried on the wind. *Canadian Journal of Veterinary Research*.

[27] Eagles D., Melville L., Weir R. (2014). Long-distance aerial dispersal modelling of *Culicoides* biting midges: case studies of incursions into Australia. *BMC Veterinary Research*.

[28] McGrath G., More S. J., O’Neill R. (2018). Hypothetical route of the introduction of Schmallenberg virus into Ireland using two complementary analyses. *Veterinary Record*.

[29] Aguilar-Vega C., Fernández-Carrión E., Sánchez-Vizcaíno J. M. (2019). The possible route of introduction of bluetongue virus serotype 3 into Sicily by windborne transportation of infected *Culicoides* spp.. *Transboundary and Emerging Diseases*.

[30] Stein A. F., Draxler R. R., Rolph G. D., Stunder B. J. B., Cohen M. D., Ngan F. (2015). NOAA’s HYSPLIT atmospheric transport and dispersion modeling system. *Bulletin of the American Meteorological Society*.

[31] Radici A., Martinetti D., Bevacqua D. (2022). Early-detection surveillance for stem rust of wheat: insights from a global epidemic network based on airborne connectivity and host phenology. *Environmental Research Letters*.

[32] Jacquet S., Huber K., Pagès N. (2016). Range expansion of the Bluetongue vector, *Culicoides imicola*, in continental France likely due to rare wind-transport events. *Scientific Reports*.

[33] Hall D. R., Torpy J., Nye R., Emma, Cowled B. (2022). Quantitative risk assessment for the introduction of lumpy skin disease virus into Australia via non-regulated pathways.

[34] Fernández-López J., Schliep K. (2019). rWind: download, edit and include wind data in ecological and evolutionary analysis. *Ecography*.

[35] Richard H., Martinetti D., Lercier D. (2023). Computing geographical networks generated by air-mass movement. *GeoHealth*.

[36] Elbers A. R. W., van den Heuvel S.-J., Meiswinkel R. (2016). Diel activity and preferred landing sites in *Culicoides* biting midges attacking Fjord horses. *Entomologia Experimentalis et Applicata*.

[37] Reynolds D. R., Chapman J. W., Harrington R. (2006). The migration of insect vectors of plant and animal viruses. *Advances in Virus Research*.

[38] Conte A., Giovannini A., Savini L., Goffredo M., Calistri P., Meiswinkel R. (2003). The effect of climate on the presence of *Culicoides imicola* in Italy. *Journal of Veterinary Medicine, Series B*.

[39] Groschupp S., Kampen H., Werner D. (2023). Occurrence of putative *Culicoides* biting midge vectors (Diptera: Ceratopogonidae) inside and outside barns in Germany and factors influencing their activity. *Parasites & Vectors*.

[40] Hendrickx G., Gilbert M., Staubach C. (2008). A wind density model to quantify the airborne spread of *Culicoides* species during north-western Europe bluetongue epidemic, 2006. *Preventive Veterinary Medicine*.

[41] Alba A., Casal J., Domingo M. (2004). Possible introduction of bluetongue into the Balearic Islands, Spain, in 2000, via air streams. *Veterinary Record*.

[42] Klausner Z., Klement E., Fattal E. (2018). Source-receptor probability of atmospheric long-distance dispersal of viruses to Israel from the eastern Mediterranean area. *Transboundary and Emerging Diseases*.

[43] Eagles D., Walker P. J., Zalucki M. P., Durr P. A. (2013). Modelling spatio-temporal patterns of long-distance *Culicoides* dispersal into northern Australia. *Preventive Veterinary Medicine*.

[44] WOAH (2023). WAHIS: World Animal Health Information System. https://wahis.woah.org/#/home.

[45] Mullen G. R., Murphree C. S. (2019). Medical and veterinary entomology. *The Japanese Journal of Veterinary Science*.

[46] Santos M. A. S., Gonzales J. R., Swanenburg M. (2023). Epizootic Hemorrhagic Disease (EHD)—systematic literature review report. *EFSA Supporting Publications*.

[47] Mills M. K., Ruder M. G., Nayduch D., Michel K., Drolet B. S. (2017). Dynamics of epizootic hemorrhagic disease virus infection within the vector, *Culicoides sonorensis* (Diptera: Ceratopogonidae). *PLOS ONE*.

[48] Wittmann E. J., Mellor P. S., Baylis M. (2002). Effect of temperature on the transmission of orbiviruses by the biting midge, *Culicoides sonorensis*. *Medical and Veterinary Entomology*.

[49] Robin X., Turck N., Hainard A. (2023). pROC: display and analyze ROC curves. https://xrobin.github.io/pROC/.

[50] Team R. C. (2022). R: a language and environment for statistical computing. https://www.R-project.org/.

[51] Greiner M., Pfeiffer D., Smith R. D. (2000). Principles and practical application of the receiver-operating characteristic analysis for diagnostic tests. *Preventive Veterinary Medicine*.

[52] Meiswinkel R., Goffredo M., Dijkstra E. G. M., van der Ven I. J. K., Baldet T., Elbers A. (2008). Endophily in *Culicoides* associated with BTV-infected cattle in the province of Limburg, south-eastern Netherlands, 2006. *Preventive Veterinary Medicine*.

[53] Versteirt V., Balenghien T., Tack W., Wint W. (2017). A first estimation of *Culicoides imicola* and *Culicoides obsoletus*/*Culicoides scoticus* seasonality and abundance in Europe. *EFSA Supporting Publications*.

[54] Wilson A. J., Mellor P. S. (2009). Bluetongue in Europe: past, present and future. *Philosophical Transactions of the Royal Society B: Biological Sciences*.

[55] MASA (2023). Instruction technique DGAL/SDSBEA/2023-724. https://info.agriculture.gouv.fr/gedei/site/bo-agri/instruction-2023-724.

[56] ESA (2023). Bulletins hebdomadaires de veille sanitaire internationale du 21/11/2023. https://www.plateforme-esa.fr/fr/bulletins-hebdomadaires-de-veille-sanitaire-internationale-du-21-11-2023.

[57] Tuel A., Eltahir E. A. B. (2020). Why is the Mediterranean a climate change hot spot?. *Journal of Climate*.

[58] Tabachnick W. J. (2010). Challenges in predicting climate and environmental effects on vector-borne disease episystems in a changing world. *Journal of Experimental Biology*.

