# Supplementary material for: Assessing the Risk of Windborne Dispersal of Culicoides Midges in Emerging Epizootic Hemorrhagic Disease Virus Outbreaks in France
Source: Transbound Emerg Dis. 2024 Jun 28;2024:5571195. doi: 10.1155/2024/5571195 (PMC12017055; doi:10.1155/2024/5571195)

**Assessing the risk of windborne dispersal of Culicoides midges in emerging epizootic hemorrhagic disease virus outbreaks in France.**

Supplement Material

*Figure S1: (A) Limits of full study grid encompassing the European countries, the North of Africa and the Balkans. (B) Study grid for France divided into grid cells of resolution 0.25 (~25 x 25 km) below ground level (sea/lakes). Atmospheric simulations were initiated from centroids of source grid cells.*


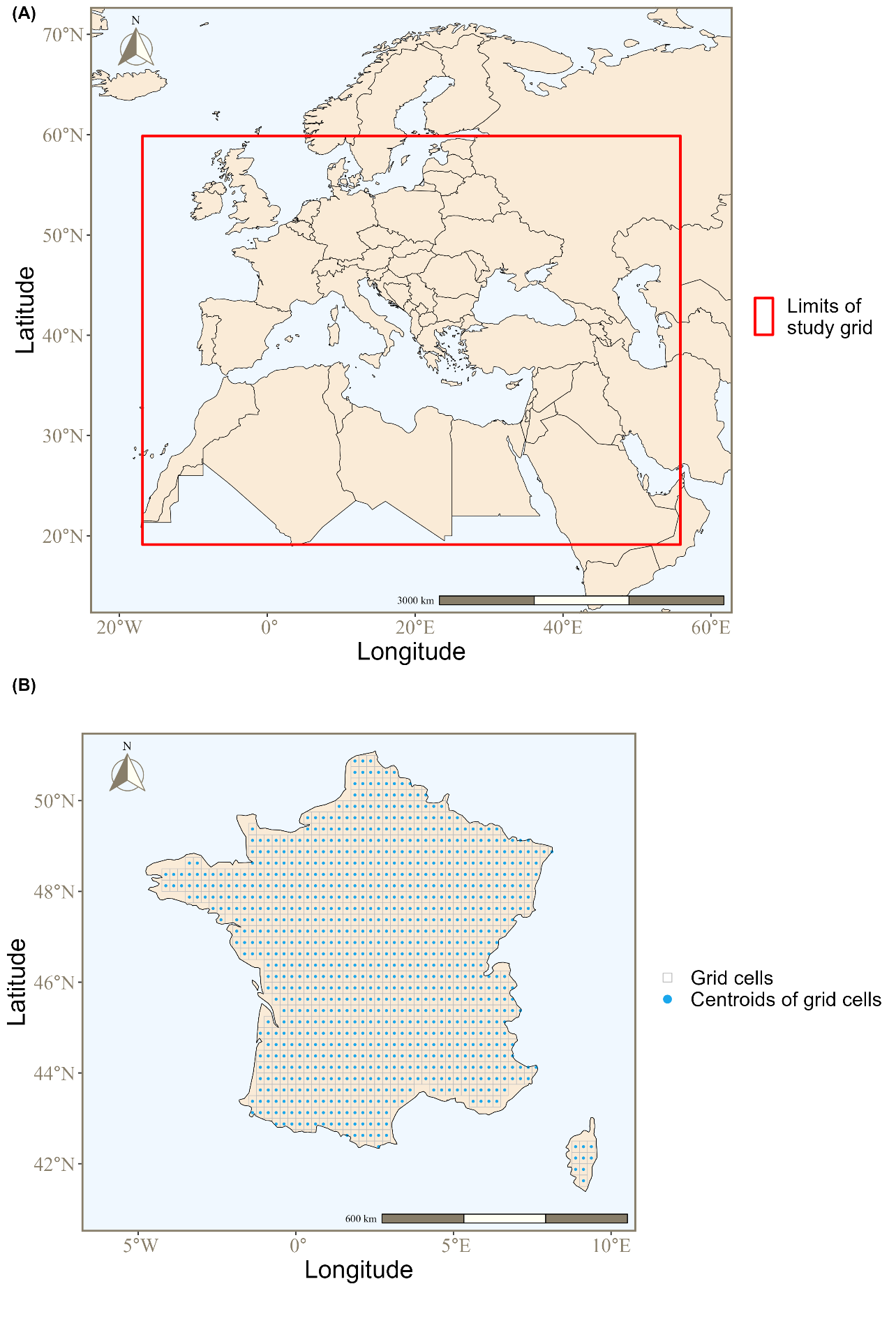


*Figure S2: Number of grid cells* $j$ *in France (continental France and Corsica) distributed for each risk interval of* $H_{j (Z_{1},T_{5})}$ *(scenario 1)*


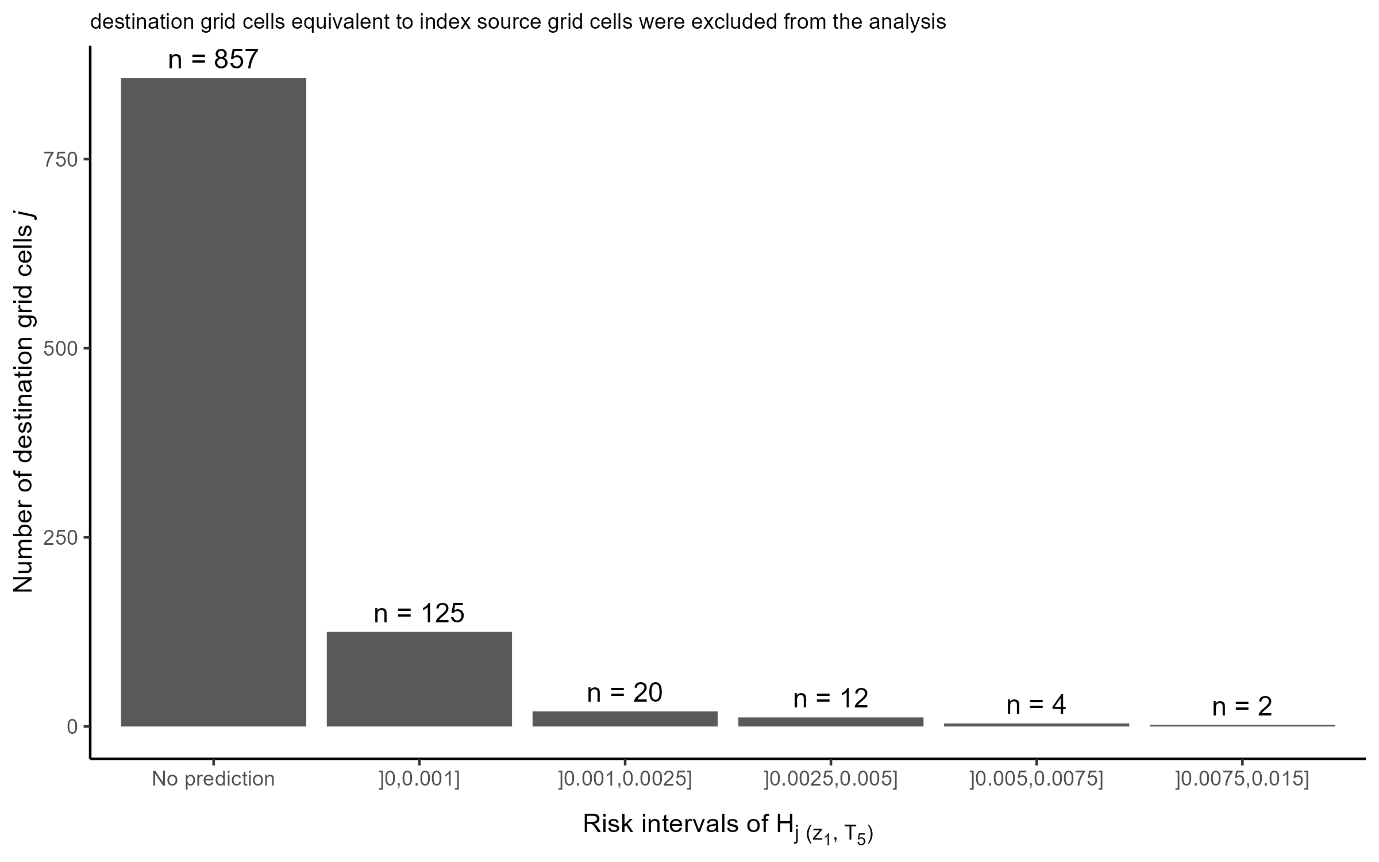


*Figure S3: Impact of time period on Scenario 1 outputs:* ***(A)*** *Spatial distribution of* $H_{j (Z_{1},T_{1})}$ *, the daily probability averaged by week (*$T_{1}$*) between week 37 and week 41, including the additional week W42 (mid-September to late-October), and* ***(B)*** *Comparison between the spatial distributions of* ***(B.1)***$H_{j (Z_{1}, T_{5})}$ *, the daily probability averaged over 5 weeks (* $T_{5}$*- from W37 to W41; from mid-September to mid-October) and* ***(B.2)*** $H_{j (Z_{1}, T_{6})}$*the daily probability averaged over the 6 weeks (*$T_{6}$*) (* $T_{6}$*- from W37 to W42; from mid-September to late-October).* $H_{j (Z_{1},T_{1})}$*,* $H_{j (Z_{1}, T_{5})}$*and* $H_{j (Z_{1}, T_{6})}$ *are all computed here considering the index source zone* $z_{1}$ *(the first 3 EHDV outbreaks started in France in week 36) and the meteorological conditions of 2023.*


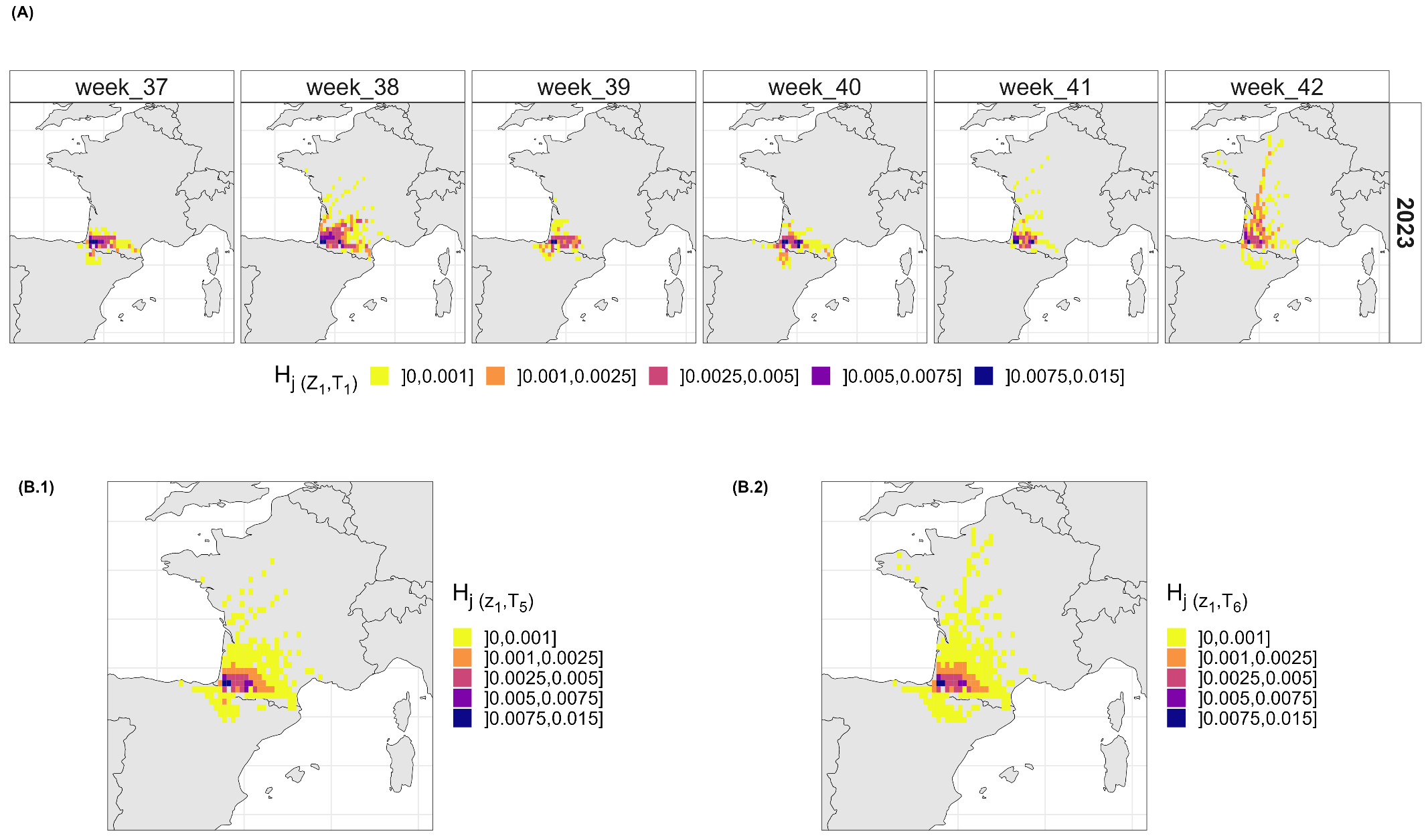


*Figure S4: Impact of time period on Scenario 2 outputs:* ***(A)*** *Spatial distribution of* $H_{j \left( Z_{2},T_{1} \right)}$ *, the daily probability averaged by week (*$T_{1}$*) of long-distance dispersal from W11 to W15, including the additional week W16 (from mid-March to late-April), and* ***(B)*** *Comparison between the spatial distributions of* ***(B.1)***$H_{j \left( Z_{2},T_{5} \right)}$ *, the daily probability averaged over 5 weeks (* $T_{5}$*- from W11 to W15; from mid-March to mid-April) and* ***(B.2)***$H_{j \left( Z_{2},T_{6} \right)}$ *, the daily probability averaged over 6 weeks (*$T_{6}$*- from W11 to W16; from mid-March to late-April).* $H_{j \left( Z_{2},T_{1} \right)}$*,* $H_{j \left( Z_{2},T_{5} \right)}$*,* $H_{j \left( Z_{2},T_{6} \right)}$ *are all computed here considering the secondary source* $Z_{2}$ *(the whole EHDV-infected area at early December 2023 in France) and the 4-year period 2020-2023.*


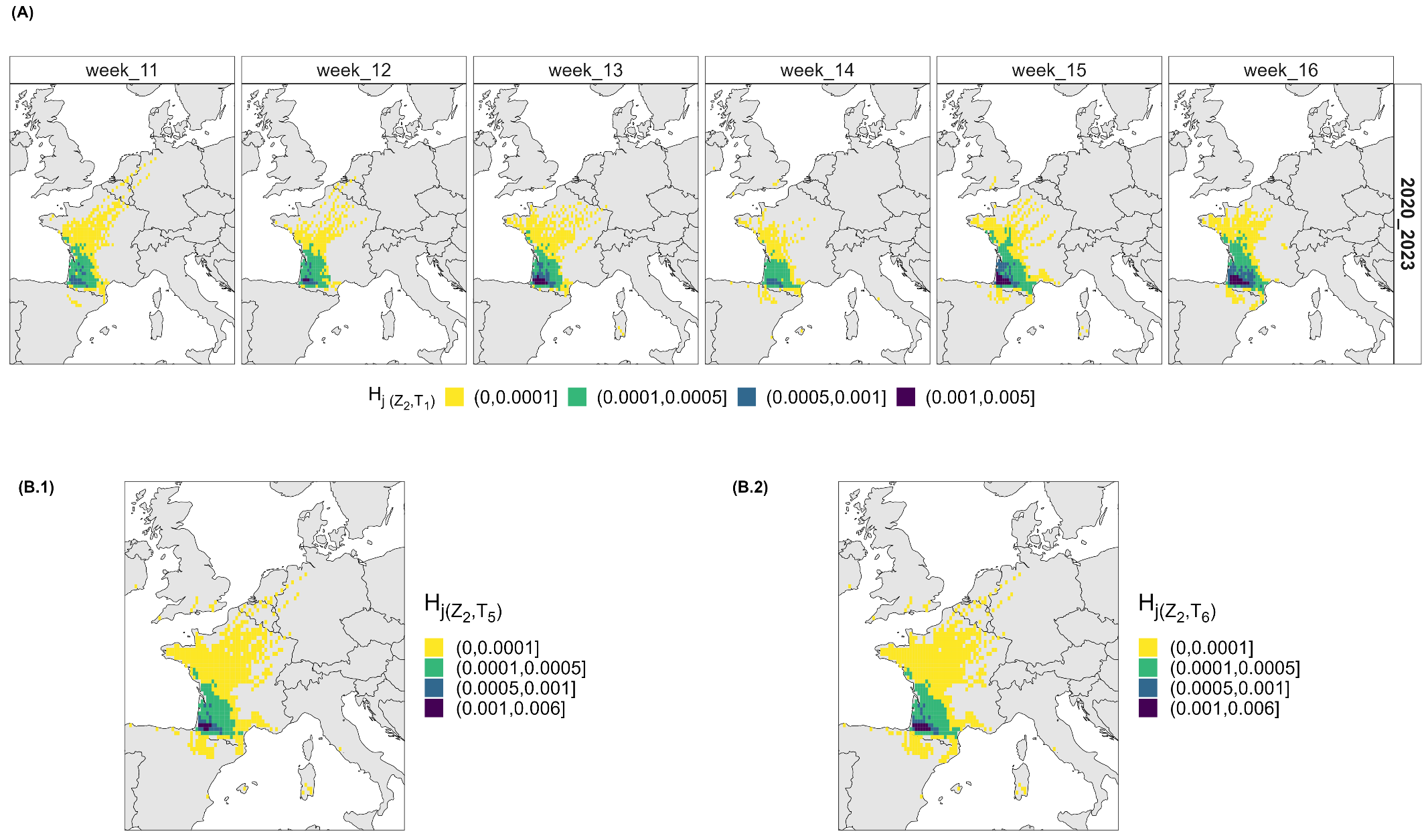

Supplement: Supplementary Materials — Figure S1 shows the spatial limits of the full study grid (A) and the division into 0.25° grid cells for France (B); Figure S2 shows the number of grid cells in France in each risk interval (scenario 1); Figures S3 and S4 illustrate the impact of adding 1 week to the time period (6 weeks) on risk maps for scenarios 1 and 2, respectively. [file 5571195.f1.docx]
